# A randomized controlled trial on the effects of goal-directed therapy on the inflammatory response open abdominal aortic aneurysm repair

**DOI:** 10.1186/s13054-015-0974-x

**Published:** 2015-06-10

**Authors:** Duane J. Funk, Kent T. HayGlass, Joshua Koulack, Greg Harding, April Boyd, Ryan Brinkman

**Affiliations:** Department of Anesthesia, University of Manitoba, 2nd Floor Harry Medovy House, 671 William Avenue, R3E 0Z2 Winnipeg, MB Canada

## Abstract

**Introduction:**

Goal-directed therapy (GDT) has been shown in numerous studies to decrease perioperative morbidity and mortality. The mechanism of benefit of GDT, however, has not been clearly elucidated. Targeted resuscitation of the vascular endothelium with GDT might alter the postoperative inflammatory response and be responsible for the decreased complications with this therapy.

**Methods:**

This trial was registered at ClinicalTrials.gov as NCT01681251. Forty patients undergoing elective open repair of their abdominal aortic aneurysm, 18 years of age and older, were randomized to an interventional arm with GDT targeting stroke volume variation with an arterial pulse contour cardiac output monitor, or control, where fluid therapy was administered at the discretion of the attending anesthesiologist. We measured levels of several inflammatory cytokines (C-reactive protein, Pentraxin 3, suppressor of tumorgenicity--2, interleukin-1 receptor antagonist, and tumor necrosis factor receptor-III) preoperatively and at several postoperative time points to determine if there was a difference in inflammatory response. We also assessed each group for a composite of postoperative complications.

**Results:**

Twenty patients were randomized to GDT and twenty were randomized to control. Length of stay was not different between groups. Intervention patients received less crystalloid and more colloid. At the end of the study, intervention patients had a higher cardiac index (3.4 ± 0.5 vs. 2.5 ± 0.7 l/minute per m^2^, *p* < 0.01) and stroke volume index (50.1 ± 7.4 vs. 38.1 ± 9.8 ml/m^2^, *p* < 0.01) than controls. There were significantly fewer complications in the intervention than control group (28 vs. 12, *p* = 0.02). The length of hospital and ICU stay did not differ between groups. There was no difference in the levels of inflammatory cytokines between groups.

**Conclusions:**

Despite being associated with fewer complications and improved hemodynamics, there was no difference in the inflammatory response of patients treated with GDT. This suggests that the clinical benefit of GDT occurs in spite of a similar inflammatory burden. Further work needs to be performed to delineate the mechanism of benefit of GDT.

**Trial registration:**

ClinicalTrials.gov Identifier: NCT01681251. Registered 18 May 2011.

## Introduction

The delivery of intravenous fluids to surgical patients is one of the most important aspects in the delivery of anesthetic care and has undergone several paradigm shifts over the past 60 years [1–4]. The goal-directed therapy (GDT) method of fluid administration relies on the utilization of minimally invasive cardiac output monitoring to tailor fluid administration to a maximal cardiac output or other reliable markers of preload such as stroke volume variation (SVV) or pulse pressure variation (PPV) [5]. In aggregate, the studies performed to date, predominantly in patients undergoing gastrointestinal surgery, have demonstrated that the utilization of GDT decreases morbidity, mortality and both hospital and ICU length of stay [6–11]. The benefit of GDT in vascular surgery patients has been less robust, presumably due to the higher rate of cardiovascular complications in this patient population [12, 13]. The mechanism of benefit of GDT, however, has been examined in only a few studies [9, 14–16]. There has been a suggestion by some that GDT reduces gut mucosal hypoperfusion and this may result in a less robust inflammatory response to surgery [17, 18].

It is possible that the benefit of GDT results from improved resuscitation of the endothelium, which in turn could be associated with decreases in the inflammatory response seen after surgery. This improved endothelial resuscitation may be associated with decreased vascular permeability resulting in less tissue edema and less tissue hypoxia which may lead to decreased postoperative organ dysfunction [19].

We hypothesized that the increase in cardiac index (CI) and decrease in postoperative complications that has been demonstrated in previous trials of GDT would be associated with decreases in inflammatory biomarkers. To that end we designed a trial to determine if GDT is associated with lower levels of inflammatory biomarkers.

In this study, we randomized 40 patients presenting for elective open repair of abdominal aortic aneurysms (AAA) to receive fluid administration based on either a GDT approach or a control method (fluid administered based on static preload parameters and traditional hemodynamics) and measured the levels several pro- and anti-inflammatory cytokines in the perioperative period. We also assessed each group for a composite of postoperative complications. Our hypothesis was that patients in the GDT group would have fewer postoperative complications and lower levels of inflammatory biomarkers in the postoperative period.

## Methods

This trial was registered at ClinicalTrials.gov as NCT01681251. After approval from the University of Manitoba Research Ethics Board, we approached all patients over the age of 18 years presenting for elective open repair of their AAA. Written, informed consent was obtained from all patients. Patients were excluded from the trial if they had any of the following: age over 80 years, weight greater than 120 kg, known or suspected aortic insufficiency, renal dysfunction (serum creatinine >150 μmol/l), active congestive heart failure, or atrial fibrillation. The weight, aortic insufficiency and atrial fibrillation exclusion criteria were included as the minimally invasive cardiac output monitor we used (FloTrac Vigeleo system; Edwards LifeSciences, Irvine, CA, USA) was inaccurate in these conditions. Patients with pre-existing renal dysfunction were excluded as they might have adverse renal outcomes from the colloid therapy utilized in our GDT protocol.

Patients were randomized to either the intervention or control group by way of a sealed envelope. Anesthetic technique was at the discretion of the attending anesthesiologist, and at our institution consists of general anesthesia, with the placement of a thoracic epidural catheter prior to induction for postoperative analgesia. The only difference in anesthetic technique between the two groups was with respect to fluid administration (see below). Standard Canadian Anesthesiologists Society monitors with the addition of an arterial line, and central venous access with an 8.5 Fr cordis were placed in all patients. In addition, the FloTrac/Vigeleo minimally invasive cardiac output monitor was utilized in all patients.

Patients were mechanically ventilated with 8 cm^3^/kg tidal volume based on ideal body weight (IBW) with respiratory rate adjusted to achieve an end-tidal CO_2_ level of 35–40 mmHg. Positive end-expiratory pressure was set at 5 cmH_2_O. Fraction of inspired O_2_ was titrated to maintain an oxygen saturation of >95 %. These specific ventilator settings were utilized to improve the accuracy of SVV determination, which was our trigger for fluid administration.

In the intervention group, patients received a background crystalloid infusion of 3 cm^3^/kg IBW of lactated Ringers solution. In the intervention group, if the SVV became greater than 13 %, 250 ml 130/0.4 hydroxyethyl starch (HES) solution (Voluven, Fresenius Kabi, Bad Homburg, Germany) was administered, and repeated until the SVV became less than 13 %. This was done up to a maximum of 55 cm^3^/kg 130/0.4 HES, and at this point further bolus fluid was changed to lactated Ringers. Of note, no patients reached this maximum limit of colloid. If the SVV was less than 13 % and CI was less than 2.2 l/minute per m^2^ inotropic therapy was started (typically norepinephrine). If SVV was less than 13 % and CI was >2.2 l/minute per m^2^ and mean arterial pressure (MAP) was less than 60 mmHg, phenylephrine by infusion was administered. Prior to aortic cross clamp removal in the intervention group, the attending anesthesiologist was allowed to administer a fluid bolus even if the SVV was <13 % in order to reduce hemodynamic instability associated with cross clamp removal.

Anesthesiologists treating patients in the control group did not have the CI or SVV information available to them from the FloTrac sensor. This information was covered on the monitor by way of an opaque index card. Control group patients had fluid administered to them at the discretion of the attending anesthesiologist, with the only stipulation being that colloid administration would be exclusively Voluven. Our institution has no protocol for GDT, and fluid in these patients is typically administered based on static preload variables (i.e., central venous pressure (CVP)) or low MAP.

Postoperative fluid administration and hemodynamic support was not protocolized and was at the discretion of the attending surgeon. This typically involved a baseline crystalloid infusion based on body weight, with a gradual transition to enteral nutrition at the surgeon’s discretion.

Autologous blood was collected and returned to the patient via cell saver, and allogenic blood was administered if there was no cell saver blood available and hemoglobin was less than 90 g/l. The transfusion of platelets, plasma and other coagulation factors was at the discretion of the attending anesthesiologists.

Intra-operative hemodynamic data (including heart rate, MAP, cardiac output, CI, SVV, CVP, and end-tidal CO_2_) were collected at 60 Hz by TrendFace Solo software (iExcellence, Wildau, Germany). The per-second values were meaned to obtain data for every minute of the case, performed off-line using Microsoft Excel (Redmond, WA, USA). Intra-operative data collected included operative duration, aortic cross clamp time, fluid administration (crystalloid, colloid, blood products), and fluid losses (blood and urine output).

Blood samples were analyzed preoperatively (baseline), immediately postoperatively, 6 hours postoperatively and 24 hours postoperatively by enzyme-linked immunosorbent assay (ELISA) for the following biomarkers associated with inflammation: interleukin (IL)-10, IL-6, Pentraxin-3 (PTX3), C-reactive protein (CRP), suppressor of tumorgenicity (ST)-2, macrophage chemotactic protein 1 (MCP-1/CCL2), IL-1-receptor antagonist (IL-1Ra), and soluble tumor necrosis factor receptor II (sTNFR-II).

Cytokines were analyzed by the following method. Analyte levels in cryopreserved serum were determined using MesoScale Discovery (MSD, Gaithersburg, MD, USA) electrochemiluminescence detection to quantify binding events on patterned arrays using minor modifications of the manufacturer’s protocol. To provide uniformity in comparing data between different lots, constant internal lab standards (purchased from Peprotech, Rocky Hill, NJ, USA, and R and D Systems, Minneapolis, MN USA) were established and used throughout the study. Briefly, samples and standards were incubated on singleplex MSD plates for 3 hours (instead of 2 hours) and the plate incubated with detection antibody for 3 hours (instead of 2 hours) before wash. All other steps were as per manufacturer’s recommendations. Analysis was on a SECTOR™ 2400 instrument (MSD). The operator was blind to the nature of all samples during processing, with subsequent statistical analysis also performed independently. Interassay variation was generally 4–10 %. There were not always sufficient samples obtained for each individual to be quantified for each analyte at each time point. Assays for which MSD plates were not available were performed by ultrasensitive ELISA as described previously [20, 21]. Briefly, titrations of four two-fold dilutions of each serum were assessed with reagents from BioLegend (San Diego, CA, USA) as described. Inter-assay ELISA variability was generally <10 %.

A blinded assessor determined postoperative complications. These included myocardial infarction, pneumonia, sepsis and septic shock, acute kidney injury, supraventricular dysrhythmia, ischemic gut, or ICU admission. All complications were adjudicated based on standard criteria [22–25].

### Statistical analysis

Based on patient data from open repair of AAA at our institution, average length of stay (LOS) for this procedure was 7 ± 3 days. We hypothesized that patients undergoing GDT at our institution could expect a 20 % decrease in their LOS. With an α level of 0.05 and a β of 0.8, we calculated our total sample size to be 40 patients. We chose LOS to power our study, as we did not know the inflammatory biomarker levels that may result from this surgery, nor what difference to expect in the intervention and control groups based on our intervention.

Statistical analysis was performed using GraphPad Prism version 6.0 (GraphPad Software Inc., La Jolla, CA, USA). Categorical variables were analyzed with the Fisher’s exact test. Between-group continuous variables were analyzed with a student’s *t*-test. The Kolmorgov-Smirnov test was performed to assess for normality. Cytokine responses were analyzed between groups utilizing repeated measures analysis of variance. Results are expressed as mean ± SD, and results were considered statistically significant if *p* < 0.05.

## Results

Baseline demographic and laboratory data are presented in Table 1. Patients in the control group were of similar age to those in the intervention group (67.9 ± 8.9 years vs. 70.6 ± 9.8 years, *p* = 0.368). There were 15 males in the control group and 12 in the intervention group (*p* = 0.31). Medical co-morbidities including diabetes, hypertension, hyperlipidemia, and ischemic heart disease were also similar between groups. The number of patients who had a previous myocardial infarction, coronary artery bypass grafting or coronary stent placement was also similar between groups. As such, the morbidity score of the Portsmouth Physiologic and Operative Severity Score for the enUmeration of Mortality and Morbidity (P-POSSUM) scores were similar between groups (63.4 ± 20.6 vs. 63.8 ± 22.8, *p* = 0.95).

**Table 1:** Patient demographics and pre-operative data

	Control group *n* = 20	Intervention group *n* = 20
Gender (male to female)	15:5	12:8
Age (years)	67 ± 8	70 ± 9
Weight (kg)	78 ± 17	81 ± 16
Hypertension	13	14
Diabetes	2	3
Hyperlipidemia	10	12
Smoker	11	7
COPD	7	11
Ischemic heart disease	8	8
Myocardial infarction	7	8
CABG	2	3
Stent	3	3
Ejection fraction (%)	55 ± 7	53 ± 11
Hemoglobin (g/l)	143 ± 15	134 ± 16
Creatinine (mmol/l)	87 ± 27	100 ± 34
eGFR (ml/minute)	83 ± 32	69 ± 29
P-POSSUM		
Morbidity rate (%)	63 ± 20	63 ± 22
Mortality rate (%)	8 ± 8	9 ± 10

Values are shown as n, unless otherwise indicated. *COPD* chronic obstructive pulmonary disease. *CABG* coronary artery bypass grafting, *eGFR* estimated glomerular filtration rate (Cockroft-Gault formula), *P-POSSUM* Portsmouth Physiologic and Operative Severity Score for the enUmeration of Mortality and Morbidity

Despite being powered for a reduction in hospital LOS, there was no difference between intervention and control groups in this parameter (8 (6–12) vs. 8 (7–13) days; *p* = 0.73).

There were no baseline differences between groups in the traditional hemodynamic parameters of heart rate, MAP, and CVP (Table 3). Cardiac index (2.5 ± 0.4 vs 2.5 ± 0.7 l/minute per m^2^, *p* = 0.94) and stroke volume index (43.8 ± 8.7 vs. 38.1 ± 9.8 ml/m^2^, *p* = 0.29) at baseline were also similar between the control and intervention groups, respectively.

**Table 2:** Intra-operative and post-operative data

	Control group *n* = 20	Intervention group *n* = 20	*p*
Intra-operative data			
Surgery duration (minutes)	228 (210–243)	210 (175–246)	0.40
Aortic cross clamp time (minutes)	50 (38–69)	52 (40–64)	0.93
Vasopressors	15	19	0.08
Estimated blood loss (ml)	725 (462–1,188)	925 (500–1,425)	0.53
Crystalloid (ml)	2,050 (1,200–2,650)	1,650 (1,050–2,088)	0.03
Colloid (ml)	500 (500–675)	1,000 (750–1,250)	<0.001
Urine output (ml)	226 (178–322)	195 (94–315)	0.29
Post-operative data			
Total crystalloid (l)	13.4 ± 6.8	11.8 ± 4.4	0.79
Total colloid (ml)	595 ± 366	1,298 ± 667	<0.001
Enteral nutrition (days)	3 (2–4)	3 (2–4)	0.44
Full diet (days)	5 (4–7)	4 (3–6)	0.43
Hospital LOS (days)	8 (6–12)	8 (7–13)	0.73

Patients in the intervention group received significantly more colloid and less crystalloid than those in the control group. Values for fluids, operative, aortic cross clamp times, enteral nutrition, full diet and hospital length of stay are shown as median (interquartile range). Other values are shown as mean ± SD. *LOS* length of stay

Surgical duration and aortic cross clamp times were similar between groups 228 (210-243) vs. 210 (174-246) minutes for surgical duration, and 50 (38-69) vs. 52 (40-64) minutes for aortic cross clamp time for control and intervention groups, respectively; see Table 2). There was a trend towards more vasopressor administration in the intervention group (19 vs. 15 patients, *p* = 0.08; Fisher’s exact test; Table 2). Estimated blood loss, cell saver blood loss and intraoperative urine output were also similar between groups (Table 2).

With respect to fluid administration, there was more crystalloid administration in the control versus intervention group (Table 2). The patients in the intervention group received more colloid (both on an ml and ml/kg basis) than those in the control group (3.5 ± 1.5 vs. 2.0 ± 1.3 ml/kg, *p* < 0.01).

With the increased colloid administration, the patients in the intervention group at the end of the study had significantly higher CI and stroke volume index (SVI) (Table 3). The SVV was also significantly lower in the intervention group. The MAP, heart rate and CVP were all similar between groups at the end of the study.

**Table 3:** Intraoperative hemodynamics

Parameter	Control group *n* = 20	Intervention group *n* = 20	*p*
MAP (mmHg)			
Beginning of study	76 ± 8	78.2 ± 11	0.50
End of study	78 ± 11	79.6 ± 7	0.63
HR (bpm)			
Beginning of study	58 ± 10	63 ± 9	0.12
End of study	67 ± 10	69 ± 11	0.65
CVP (mmHg)			
Beginning of study	9.3 ± 3	10.4 ± 5	0.48
End of study	10.3 ± 4	11.1 ± 3	0.58
CI (l/minute per m^2^)			
Beginning of study	2.5 ± 0.4	2.5 ± 0.3	0.94
End of study	2.5 ± 0.7	3.4 ± 0.5	<0.0001
SVV			
Beginning of study	10.0 ± 5	9.6 ± 2	0.79
End of study	12.1 ± 5	5.6 ± 2	<0.001
SVI (ml/m^2^)			
Beginning of study	43 ± 8	40 ± 6	0.29
End of study	38 ± 9	50 ± 7	<0.001

*CI* cardiac index, *CVP* central venous pressure, *HR* heart rate, *MAP* mean arterial pressure, *SVI* stroke volume index, *SVV* stroke volume variation

In the postoperative period (postoperative days 1–7) there was no difference between groups with respect to crystalloid or colloid administration, or red blood cell transfusion. On average, patients tolerated their first enteral diet and full diet on the same postoperative day.

The composite outcome of perioperative complications occurred more frequently in the control group than the intervention group, and this was statistically significant (28 vs. 12, *p* = 0.02, Fisher’s exact test; Table 4).

**Table 4:** Postoperative complications

	Control group *n* = 20	Intervention group *n* = 20	*p*
Myocardial infarction	3	1	
Pneumonia	1	1	
Respiratory failure	1	0	
Sepsis	1	0	
Rhabdomyolysis	1	0	
Acute kidney injury	4	4	
Dysrhythmia	3	2	
Bleeding	2	1	
Ischemic gut	1	0	
Delirium	3	2	
ICU admission	6	1	
Death	2	0	
Total	28	12	0.02

**Fig. 1:**
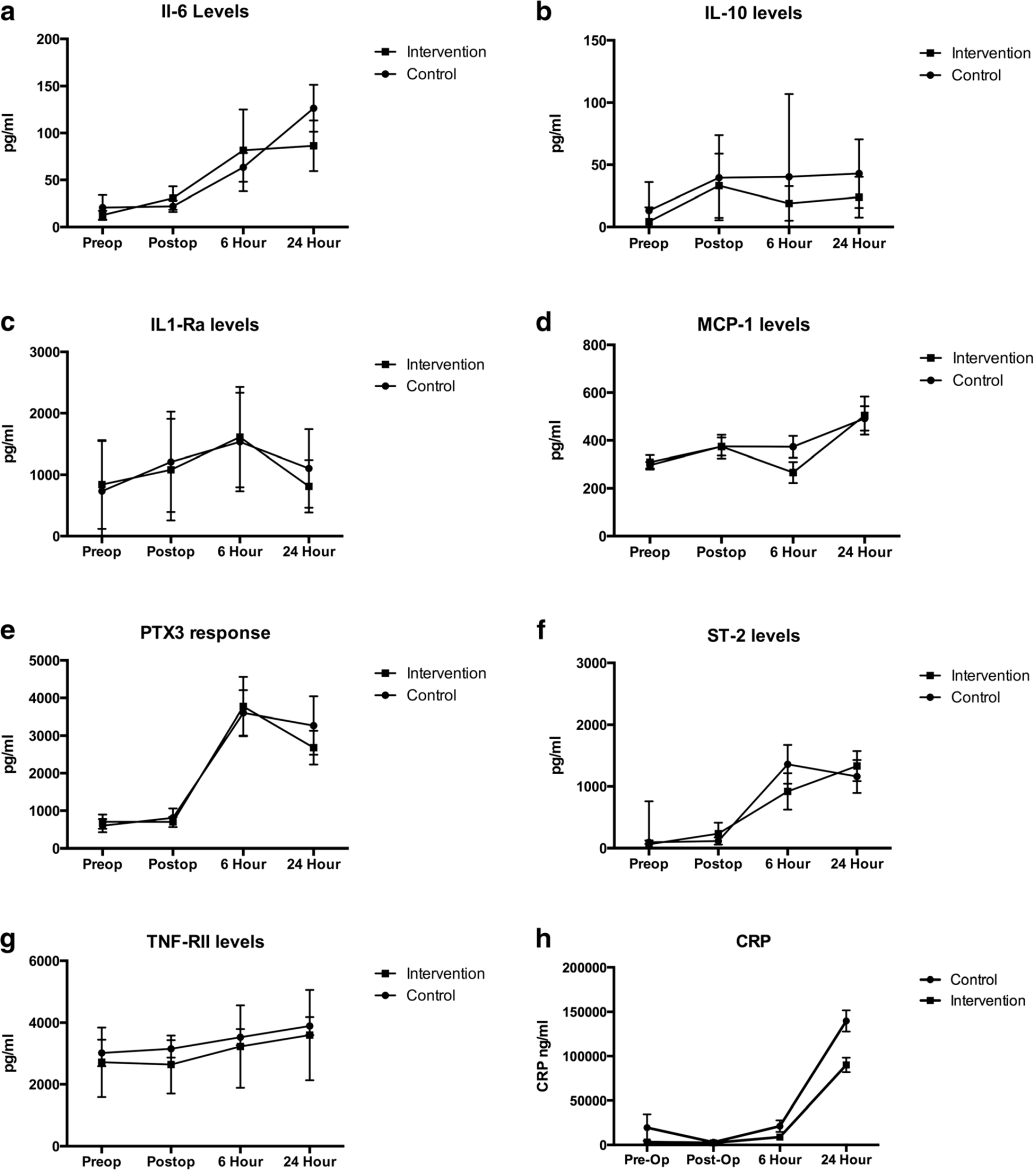
Cytokine levels over time. **a** Interleukin-6 (*Il-6*); **b** interleukin-10 (*IL-10*); **c** interleukin-1-receptor antagonist (*IL1-Ra*); **d** macrophage chemotactic protein-1 (*MCP-1*); **e** Pentraxin 3 (*PTX3*); **f** suppressor of tumorgenicity-2 (also known as interleukin receptor-1-like 1) (*ST-2*); **g** tumor necrosis factor receptor type II (*TNF-RII*); **h** C-reactive protein (*CRP*). There was no difference between the control and intervention groups (group versus time interaction, repeated measures analysis of variance, all *p* > 0.05)

All of the biomarkers measured demonstrated a statistically significant increase in levels from baseline to the 24-hour period (see Fig. 1, all *p* < 0.01). This result indicates that the biomarkers that we chose to measure were elevated in response to surgical stress, and that the time course of cytokine response was adequately captured with our sampling method.

With the exception of CRP, there was no group versus time difference in inflammatory biomarker levels between the control and intervention groups (see Fig. 1).

## Discussion

Our study, like many others, demonstrated that the utilization of GDT in patients undergoing high-risk surgery results in an increased CI and SVI when compared to controls. This increase in CI was due to an increase in the amount of colloid administered in the intervention group. This increase in CI was also associated with a decrease in postoperative complications. Despite this increase in CI and decrease in postoperative complications, GDT was not associated with a decreased LOS nor a reduction in the level of inflammatory biomarkers (with the exception of CRP). Our hypothesis was that the improved CI seen in GDT patients was due to improved resuscitation of the endothelium and would be manifest by decreases in inflammatory biomarkers.

The lack of a difference in LOS could be due to many factors not related to the hemodynamic improvements seen in our intervention group. For example, patients requiring home-care services may be delayed in their discharge from hospital. Fitness for discharge is likely a better parameter to examine in this situation.

We chose a broad panel of both pro- and inflammatory biomarkers that are expressed in periods of surgical or medical stress to determine if GDT resulted in a diminished perioperative inflammatory response. The surgical stimulus resulted in a significant inflammatory response as demonstrated by the fact that cytokines measured at the immediate postoperative time period were significantly greater than baseline (all *p* < 0.05, data not shown).

Renal cells in response to inflammatory stimuli secrete MCP-1, and MCP-1 levels are elevated in glomerulonephritis and diabetic nephropathy [26, 27]. MCP-1 is also elevated in patients with atherosclerotic disease [28, 29]. PTX3 is part of the humoral arm of innate immunity, and is in the same family as CRP. Increased levels of PTX3 have been seen in ischemia-reperfusion injury as well as patients with septic shock and acute kidney injury.

Traditional fluid administration to patients presenting for high-risk surgery relied on the use of static parameters such as CVP, MAP, and urine output. There are several studies that have shown that using these parameters to gauge fluid administration leaves patients effectively hypovolemic with decreased CI [8, 10, 11, 30]. This decrease in CI has been shown to be associated with gut mucosal hypoperfusion and this may lead to an increase in the inflammatory response seen after surgery [31–33].

There are few papers that have studied the inflammatory response to GDT. Noblett et al. [9] randomized patients undergoing elective colonic resection to GDT or control using esophageal Doppler as their cardiac output monitor. Similar to our study, they demonstrated an increase in CI in the intervention group and a reduced incidence of complications in the postoperative period. They measured IL-6 levels at similar time points to our study and found that the peak IL-6 levels (at the 6-hour time point) were significantly higher in the control group, suggesting that there was less inflammation in their GDT group [9]. Measured at the same time point (6 hours postoperatively), the levels of IL-6 in our study (81 ± 144 pg/ml) were lower than those of Noblett (369 pg/ml). It is unclear why a difference was demonstrated in their study and not ours, and why the levels of cytokines were almost fivefold higher in their study. Differences in assay techniques and sensitivity could explain the differences between our studies.

In a mouse endotoxin model, levels of tumor necrosis factor (TNF)-α were lower in animals given dopexamine to improve global blood flow when compared with controls [15]. This decrease in TNF-α in the dopexamine group occurred despite similar SVI.

Jhanji et al. also looked at inflammatory biomarkers in a GDT trial in gastrointestinal surgery patients [16]. They demonstrated increased oxygen delivery and improved sublingual and cutaneous microvascular flow in patients treated with stroke volume optimization and dopexamine administration. They also found no difference in inflammatory biomarkers (IL-6, IL-8, IL-1β, TNF-α, and ICAM-1) between the dopexamine-treated group and a control group who had their fluids titrated based on CVP alone.

The only other paper that examined the inflammatory response to GDT was from Rivers et al. [34]. In this study, Rivers looked at the cytokine response in patients from his seminal paper on GDT for septic shock [35]. In this study he found that the levels of IL1-Ra, TNF and IL-6 were higher in the patients that underwent GDT for their septic shock. This therapy included the aggressive administration of fluids, vasopressors, red blood cell transfusion and treatment with inotropes. Levels of cytokines in this study were approximately 10-fold higher than in our study, indicating a much higher inflammatory burden in these critically ill patients.

It is difficult to compare our results with those in the Rivers study as his patients had an ongoing stimulus for the inflammatory response (their ongoing infection that caused the septic shock), whereas at the end of surgery the inflammatory response in our patients was removed.

Our results are, however, consistent with those of Jhanji et al. looking at the role of the inflammatory response to GDT [16].

Furthermore, it is difficult to examine only one cytokine and make broad comments about the overall inflammatory response. The human immune system is pleiotropic and redundant and there is abundant cross regulation between the pro- and anti-inflammatory arms. We measured cytokines from both the pro- and anti-inflammatory arms, and therefore can make more robust conclusions about the overall inflammatory burden that our patients underwent.

How then do we explain the fact that patients in our GDT group had improved CI and fewer complications yet suffered a similar inflammatory burden? It is known that endothelial integrity is maintained via proteins that intercalate between cells. Endothelial cells are tightly bound together by a protein called VE-cadherin. VE-cadherin is present on the surface of endothelial cells and is responsible for preventing interstitial edema. Cytokines and other inflammatory mediators are known to disrupt these proteins, leading to increased endothelial permeability, tissue edema, cellular hypoxia and organ dysfunction [19]. Expression of the Slit protein and its cognate receptor Robo have been shown to stabilize VE-cadherin protein binding between cells, thus reducing endothelial permeability [36]. Interestingly, in animal models of acute respiratory distress syndrome, septic shock, and infection with avian influenza virus, pretreatment with Slit protein reduced disease mortality in infected animals, but did not reduce cytokine levels [37]. This suggests that vascular integrity was maintained, despite a similar inflammatory response.

A similar phenomenon could have occurred in our patients; that is, despite a similar inflammatory stress in both groups of patients (which would be expected as they underwent identical procedures), endothelial integrity was maintained in the GDT group, and this resulted in less tissue hypoxia and organ dysfunction. Our results suggest that the improved postoperative outcomes seen in GDT are not related to differences in the inflammatory response, and thus must result from a different mechanism.

Therefore, an alternative way of interpreting our results would be to conclude that, despite a similar inflammatory burden, patients in the GDT group had fewer complications.

The earliest studies of GDT examined the concept of oxygen debt in the critically ill and high-risk surgical patients [38, 39]. These studies found patients that presented to the emergency department with shock had lower oxygen delivery indices than those who survived their shock state. These early observations led to the concept of an oxygen debt that patients incur when undergoing high-risk surgery or when they are critically ill. This oxygen debt leads to tissue hypoperfusion and organ system dysfunction. Subsequent studies utilized an oxygen delivery-based protocol for fluid, blood product and inotrope administration to try and maximize oxygen delivery. Some of these studies found that targeting an increased oxygen delivery resulted in lower mortality and fewer complications [40–42]. Subsequent trials were unable to confirm these results [43–45]. Unfortunately, we did not measure oxygen consumption in our patients, so we cannot be sure if our control group incurred an oxygen debt. However, despite the differences in CI between groups at the end of the study, oxygen delivery was not significantly different between groups, thus lessening the likelihood that a significant oxygen debt played a role in the increased complication rate in the control patients.

Limitations to our study include the small size, and the inability to measure CI in the postoperative period. Further, due to logistical complications, we only measured cytokine levels to 24 hours, and there may have been a difference after this time point. However, in our pilot trial, we measured cytokine levels at 48 hours and at this time point the levels were returning to baseline. Utilizing a SVV target of 13 % has also recently come under question as being in a ‘grey zone’ of fluid responsiveness [46]. Despite this, we did demonstrate an increase in CI in our intervention group.

We also did not measure lactate in the postoperative period, which would potentially have informed us if an oxygen debt or tissue hypoperfusion were occurring.

Future studies should focus on alternative explanations for the underlying mechanism as to why patients in the intervention arm of GDT trials have reduced complications.

## Conclusion

Our study demonstrated that GDT in patients undergoing open repair of their AAA was not associated with a reduction in the inflammatory response. Future studies on the mechanism of benefit of GDT should focus on mechanisms other than alterations in the inflammatory response.

## Key messages

- GDT in high-risk vascular patients results in improved CI/SVI.
- This improvement in hemodynamics resulted in decreased complications.
- These improved outcomes occurred despite a similar magnitude in the inflammatory response to surgery.
- Future trials examining the mechanism of benefit of GDT should focus on pathways other than the inflammatory response.

### Ethics

This report describes human research. IRB contact information: Bannatyne Research Ethics Board, P126 Pathology Building, 770 Bannatyne Avenue, University of Manitoba, Winnipeg, MB, R3E 0 W3. Phone: 204 789–3255. Fax: 204 789–3414. This study was conducted with written informed consent from the study subjects. This report describes a prospective randomized clinical trial. The author states that the report includes every item in the CONSORT checklist for a prospective randomized clinical trial.

## Abbreviations

AAA: Abdominal aortic aneurysms
CI: Cardiac index
CRP: C-reactive protein
CVP: Central venous pressure
ELISA: Enzyme-linked immunosorbent assay
GDT: Goal-directed therapy
HES: Hydroxyethyl starch
IBW: Ideal body weight
IL: Interleukin
IL-1Ra: Interleukin-1-receptor antagonist
LOS: Length of stay
MAP: Mean arterial pressure
MCP-1: Macrophage chemotactic protein 1
PPV: Pulse pressure variation
PTX3: Pentraxin 3
ST: Suppressor of tumorgenicity
SVI: Stroke volume index
SVV: Stroke volume variation
sTNFR-II: Soluble tumor necrosis factor receptor II
TNF: Tumor necrosis factor
